# Firearm injury and young children: a critical review

**DOI:** 10.1186/s12887-025-06052-2

**Published:** 2025-09-24

**Authors:** Alison L. Miller, Wadad Itani, Hurley Riley, Ezekiel Medina, Odalys Arbelaez, Julia Plawker, Andrew Hashikawa, Daniel Lee, Justin Heinze, Cynthia Ewell-Foster, Rebeccah Sokol, Hsing-Fang Hsieh

**Affiliations:** 1 University of Michigan School of Public Health, Ann Arbor, USA; 2 University of Michigan School of Medicine, Pediatrics and Emergency Medicine, Ann Arbor, USA; 3 University of Michigan Institute for Firearm Injury Prevention, Ann Arbor, USA; 4 University of Michigan School of Medicine, Psychiatry, Ann Arbor, USA; 5 University of Michigan School of Social Work, Ann Arbor, USA

**Keywords:** Firearm violence, Firearm injury, Early childhood, Preschool, Toddler, Parent, Prevention

## Abstract

Firearms are the leading cause of death in children in the United States. Rates of firearm injury and death are rising among young children, ages 0–5 years, yet data-driven prevention strategies are lacking. The goal of this critical review was to provide a developmentally-informed overview of the patterns and prevalence, contexts, and impacts for all-cause firearm injury among children ages 0–5 years. We review findings from interdisciplinary, peer-reviewed studies to inform considerations for prevention strategies. We present disparities in injury and death rates in this age group, with males and non-Hispanic Black children most impacted. We review data regarding the contexts of firearm injury and death among young children; although most injuries in this age group occur at home, young children also experience firearm injury through community violence. The physical, psychological, and social impacts of injury on individual children, their families, and their communities are also reviewed. Integrating these findings, we present strategies for firearm injury prevention in this age group, informed by the bioecological systems model of development and the Haddon Matrix for injury prevention, considering factors in the child’s proximal physical and social environment and broader social context. Most research does not specifically focus on young children, resulting in limited data on firearm injuries within this age group. Consequently, we recommend that future qualitative and mixed-methods studies prioritize this developmental period, actively engaging parents and community members connected to young children. This approach will contribute to a better understanding of the nature of firearm injuries and death among young children and inform effective prevention. We also call for research-community partnerships to evaluate the impact of policy and community-based prevention initiatives on injury rates in young children. We hope that this critical review serves as an initial roadmap for researchers, public health professionals, clinicians, and anyone working with parents and young children, providing a reference for preventing avoidable injuries and deaths in the youngest members of our population.

## Background

In 2020, firearm injury became the leading cause of death for youth in the United States (US) [1]. Rates of firearm injury and death among children 0–5 years old are rising rapidly, with a 75% increase in firearm deaths in this age group from 2010 to 2020 [2]. Of firearm fatalities in children under age 15 from 2009 to 2018, 40% were among 2–4 year olds [3]. Although it has been suggested that increases may be in part due to increasing firearm ownership during the COVID-19 pandemic [4, 5], all-cause mortality in children had been rising prior to the pandemic, with firearms playing a key role [6]. Young children who sustain a firearm injury are twice as likely to die from their injuries as older youth [7]. Even if a child recovers, the psychological and health impacts of experiencing such trauma can be lifelong [8]. Yet, firearm-related injuries in young children have not received the same level of research attention as compared to older children and youth [2].

To address this gap and inform insights for prevention, we conducted a critical review [9] of key issues regarding firearm injuries among children 0–5 years of age. The goal was to describe the scope of such injuries and to characterize the context(s) in which they occur in this age group and their broader impacts, to generate recommendations for prevention. Our work is theoretically informed by Bronfenbrenner’s bioecological systems model of development, which details not only the interconnected nature of the nested systems in which children develop, but also the proximal processes in children’s immediate environments that interact with individual child (or “person”) factors to shape development [10]. Nested systems relevant for firearm injury prevention include the microsystem (e.g., child’s home environment), mesosystem (e.g., interactions among child’s caregivers in different contexts on behalf of the child), exosystem (e.g., influences not involving the child, for example parents’ friends), macrosystem (e.g., community social norms, policies), and chronosystem (e.g., historical events such as the COVID-19 pandemic). Relevant child factors include age, sex, and temperament (e.g., impulsivity) and proximal processes include parenting (e.g., supervision, storage practices). This model informed our review by guiding the design of research questions and the interpretation of the relatively limited literature on firearm-related injury in this age group to identify crucial considerations and highlight gaps regarding firearm injuries in the early childhood period.

Specifically, we used this model to inform our three research questions, with each question considering aspects of how the interconnected systems in which young children develop inform their risks for firearm-related injury. Question 1 considered the scope of the problem for this young age group and sought to identify whether broad social patterns of injury and death at the outer contextual level were present for young children, asking: what are the *patterns* of firearm injury in this population, including disparities? Question 2 sought to characterize the nature of firearm injuries in proximal contexts for young children, asking: what is known regarding *contexts* surrounding firearm injury in this population? Question 3 considered how injuries to young children in turn impacted both proximal as well as more distal contexts in the system, asking, what are the physical and psychological *impacts* of such injuries? We sought to synthesize these data to highlight *strategies for preventing* firearm injuries in young children.

We reviewed databases commonly used in public health and child development/pediatric research (PubMed, CINAHL, Scopus, PsycInfo) as well as Google Scholar for the initial review phase. We also found studies using citation chaining and snowball methods where references of highly relevant articles were searched to identify new articles. The search was guided by our research questions; that is, did papers cover the scope of firearm-related injuries for children in this age group (Question 1), did papers address the contexts of such injuries (Question 2), and did papers address impacts of such injuries (Question 3). We also identified papers that focused on prevention or intervention strategies and noted whether those would be relevant to young children, for our goal of informing prevention. Our team was composed of graduate students, researchers, and professors with expertise in public health, child development, and firearm injury prevention science. At least 2 team members reviewed each paper. We used search terms capturing firearm injury (e.g., gun violence, firearm, injury, gunshot, shooting) and pediatric populations (e.g., early childhood, child, preschool, 0–18, p(a)ediatric). We focused only on studies in the US. Studies were limited to English language and peer-reviewed. The review was conducted between December 2023 and July 2024. We focused on studies published during the previous 10 years due to the marked increase in federal funding for firearms research beginning in 2013 in response to the revision to the Dickey Amendment specifying that it did not prevent federal funding for research on firearm injury prevention, with a corresponding increase in published studies since this time [11]. Title and abstracts were reviewed first, proceeding to full papers if relevant. Given the limited number of studies and data specifically on the early childhood period, we initially considered studies including children ages 0–18 to capture samples that included young children. However, we include in our critical review only studies that either exclusively focused on children 0–5 years of age, or stratified by age with at least one age group being 0–5 year-olds, so that we could report on more granular age-based findings. Studies that did not either exclusively focus on this age range or stratify by age and include this age group as one of the strata were excluded from the final review tables.

Sections of the critical review are organized by research question, concluding with a synthesis of implications of these findings for intervention and recommendations for future research directions. We identified 129 papers that proceeded to the full review stage; of these, 71 were directly relevant to the 3 research questions, and 58 concerned prevention or intervention strategies. After conducting a full review of the 129 papers, we identified 18 papers (listed in Table 1) that specifically considered children in the 0–5 age range and directly informed our research question(s). We included 20 papers that focused on intervention or prevention strategies relevant for young children (listed in Table 2).

**Table 1:** Papers including data specific to children in focal age range (0–5 years)

Citation	Country	Study aim	Sample/data sources	Child ages	% of sample within 0–5 year age range	Relevant for research question(s)
Andrews et al. (2022)	USA	Examine changes in firearm injury mortality among youth 0–19 years from 2001 to 2019	Centers for Disease Control and Prevention youth mortality data (WISQARS); rates per 100,000	0–19 years	% and n not reported; examined proportion of deaths by intent for 0–4 year-olds	1
Bagdure et al. (2021)	USA	Examine the outcomes for children admitted to PICU with firearm injuries	PICU data for children admitted with firearm injuries (VPS; *n* = 1447 cases)	1 month-18 years	17.0% (*n* = 246) were 1 month-5 years	3
Bleyer et al. (2021)	USA	Characterize unintentional firearm mortality trends in 1–4 year-olds	Firearm-related mortality data (WONDER; WISQARS; NVDRS)	1–4 years old	100% were 1–4 years old (n not reported)	1, 2
Cheng and Burjonrappa (2022)	USA	Examine trends in overall and intent-specific firearm hospitalizations across age and race	Children involved in firearm incidents (NTDB data; *n* = 20,223)	0–18 years	1.6% (*n* = 317) were 0–2 years	1
Collings et al. (2022)	USA	Examine whether firearm injuries in children were associated with stay-at-home orders during the COVID-19 pandemic	Patients with traumatic injuries (NTDB data; *n* = 215)	< 18 years	13.4% (*n* = 29) were 0–4 years in the 2020 COVID cohort	2
Cook et al. (2019)	USA	Characterize hospital survival among gunshot wound victims *≤* 19 years	Children seen in emergency department for gunshot wound (Healthcare Cost and Utilization Project, NEDS data; *n* = 82,569)	0–19 years	2.2% (*n* = 1822) were 0–4 years	2, 3
Faulkenberry and Schaechter (2015)	USA	Characterize unintentional pediatric firearm injury events coverage in media	Unintentional firearm injury cases from Google News (*n* = 277)	0–18 years	13.7% (*n* = 38) were 1–3 years	1, 2
Holloway et al. (2023)	USA	Examine impacts of gun violence on early childhood development	Review of literature	0–18 years	Not applicable	3
Kalesan et al. (2016)	USA	Compare rates of firearm-related to vehicular-related hospitalizations	Nationwide Inpatient Sample data (age-matched sample of 4725)	0–15 years	9.8% were 0–5 years (n not reported)	1
Loder and Luster (2023)	USA	Examine fracture patterns in pediatric firearm injuries	US Firearm Injury Surveillance Study (hospital data; *n* = 711)	0–15 years	9.1% (*n* = 65) were 0–5 years	2, 3
Mitchell et al. (2019)	USA	Examine impact of witnessing gun violence on youth living in urban and nonurban areas	Survey data from Boston, Philadelphia, and rural areas of eastern Tennessee (*n* = 630)	2–17 years	20% (*n* = 126) were 2–4 years	2, 3
Nordin et al. (2018)	USA	Characterize morbidity of unintentional firearm injuries	Children with gunshot wounds (NTDB data; *n* = 7487)	0–14 years	18.3% (*n* = 1375) were 0–4 years	1, 2
Oddo et al. (2021)	USA	Examine new mental health diagnoses after nonfatal firearm injuries	Firearm injury data; Truven Health Analytics Market-Scan Multi-State Commercial and Medicaid Claims (*n* = 2178)	0–17 years	7.4% (*n* = 162) were 0–5 years	3
Price and Khubchandani (2023)	USA	Characterize fatal firearm violence in children ages 0–5 years	Firearm-related mortality data (WISQARS; *n* = 1220)	0–5 years	100% (*n* = 1220) were 0–5 years	1, 2
Prickett et al. (2019)	USA	Examine changes in firearm ownership among families with young children, 1976–2016	National Vital Statistics System; General Social Survey (nationally representative dataset of US households)	0–5 years	100% (n = not reported)	1, 2
Roberts et al. (2023)	USA	Examine trends in 2021 data on USA pediatric firearm deaths, disparities	Pediatric firearm-related mortality data (WONDER; *n* = 4752)	0–19 years	3.2% (*n* = 153) were 0–4 years	1
Swendiman et al. (2020)	USA	Characterize pediatric firearm injuries	Pediatric firearm injury data (NTDB; *n* = 36,581)	0–19 years	2.5% (*n* = 911) were 0–4 years	1, 3
Vaishnav et al. (2023)	USA	Characterize firearm fatalities in which children < 15 years unintentionally killed themselves or another child	Firearm fatality data (NVDRS Restricted Access Dataset; *n* = 279)	0–15 years	43.4% (*n* = 121) were < 5 years	1, 2, 3
Wilson et al. (2023)	USA	Examine characteristics of firearm injury deaths among 0–17 year olds	Firearm-related mortality data (NVDRS; *n* = 1262)	0–17 years	29.1% (*n* = 367) were 0–5 years	1, 2

National Violent Death Reporting System (NVDRS); National Trauma Data Bank (NTDB); Nationwide Emergency Department Sample (NEDS); Virtual Pediatric Systems (VPS); Web-based Injury Statistics Query and Reporting System (WISQARS); Wide-ranging Online Data for Epidemiologic Research (WONDER)

Research Questions: (1) *patterns* of firearm injury; (2) *contexts* surrounding firearm injury; and (3) physical and psychological *impacts* of firearm injuries

**Table 2:** Papers on firearm injury prevention strategies relevant to children 0–5 years of age, aligned with Haddon matrix

Citation	Country	Study aim	Sample	Child ages	% of sample within 0–5 year age range	Haddon matrix level of prevention
Hanratty et al. (2016)	USA	Evaluate effectiveness of a behavioral skills training program followed by in situ training	3 boys and 2 girls attended same preschool in low SES urban area (*n* = 5)	4 years	100% (*n* = 5/5) were 4 years	Host
Miltenberger et al. (2022)	USA	Evaluate effects of video self-modeling for teaching gun safety skills to children with developmental disabilities	Children diagnosed with intellectual disabilities (*n* = 5)	5–13 years	40% (*n* = 2/5) were 5 years	Host
Baruni and Miltenberger (2022)	USA	Discuss best practices for assessing and training safety skills in existing literature	N/A - review	N/A	N/A	Host
Novotny et al. (2023)	USA	Evaluate a web-based program for teaching parents to conduct behavioral skills training to prevent child gun play	Parent-child dyads in Florida and Texas (*n* = 18)	3–7 years	Not reported	Host
Crifasi et al. (2019)	USA	Study examining desirability of personalized guns	Gun owners (*n* = 21); study focused on “childproof” guns	N/A	N/A	Agent
Lee et al. (2022)	USA	Discuss prevention strategies for firearm-related injuries and deaths in children	N/A - review	N/A	N/A	Agent
Rowhani-Rahbar et al. (2016)	N/A	Systematic review of randomized and quasi-experimental controlled studies of safe firearm storage interventions	N/A - review	N/A	N/A	Proximal Physical/Social Environment
Barkin et al. (2008)	USA	Determine whether primary care practitioner intervention would affect patients’ families’ violence-prevention behaviors	Caregivers of children ages 2–11 years presenting for a well-child visit (137 pediatric practice offices)	N/A	N/A	Proximal Physical/Social Environment
Hoops and Crifasi (2019)	USA	Characterize the firearm-related anticipatory guidance practices of pediatricians-in-training	81 pediatric residents in the Mid-Atlantic region	N/A	N/A	Proximal Physical/Social Environment
Roszko et al. (2016)	N/A	Systematic review of literature on clinical firearm injury prevention screening and interventions	N/A - review	N/A	N/A	Proximal Physical/Social Environment
Garbutt et al. (2016)	USA	Determine whether parents are receptive to discussing firearm safety with their pediatrician	Parents from 13 participating practices in St. Louis, Missouri.	Median age of child with appt = 5.0 years	N/A	Proximal Physical/Social Environment
Campbell et al. (2020)	USA	Test feasibility of tablet-based firearm safety guidance	Parents from 9 pediatric practices in 15 states	0–18 + years	32% of sample was 0–3 years old (*n* = 173/543)	Proximal Physical/Social Environment
Thomas et al. (2019)	USA	Describe “Be SMART for Kids” program for talking about gun safety	N/A	N/A	N/A	Broad Social-Contextual
Ewell Foster et al. (2024)	USA	Conduct focus groups and key informant interviews to design a safe storage prevention strategy for rural families	40 residents of rural county in Midwestern USA (60% male)	N/A	N/A	Broad Social-Contextual
Hammig et al. (2024)	USA	Examine prevention messaging regarding the safe storage of firearm among media outlines when reporting on unintentional firearm injury deaths	223 reported deaths among children aged 0–11 years	0–11 years	6.7% (*n* = 15/223) of sample was 0–1 years of age	Broad Social-Contextual
Kondo et al. (2015)	USA	Review findings from studies of nature-based interventions regarding impacts on health, perceptions of safety, and crime	N/A - review	N/A	N/A	Broad Social-Contextual
Bonne et al. (2021)	USA	Review literature to provide evidence-based recommendations for community-based programs to mitigate gun violence	N/A - review (19 studies)	N/A	N/A	Broad Social-Contextual
Azad et al. (2020)	USA	Evaluate the association between state child access prevention firearm laws and pediatric firearm fatalities	N/A	Examined differences in state fatality rates in children 0–14 years	N/A	Broad Social-Contextual
Prickett et al. (2014)	USA	Investigate how state-level firearm legislation is associated with firearm ownership and storage among families with preschool-aged children	Approximately 8100 families from a nationally representative survey of children born in 2001	Parents of children 4 years of age	100% were 4 years	Broad Social-Contextual
Miller et al. (2022)	USA	Examine whether child access prevention negligent storage laws affect firearm storage	2950 gun owners	N/A	N/A	Broad Social-Contextual

## Research question 1: patterns of firearm injuries and deaths among children 0–5 years

### Rates and intent

Firearm-related injury intent is generally classified as unintentional, assault/homicide, suicide/self-harm, and legal intervention, with data more reliably available for fatal compared to non-fatal shootings [12]. Only a few studies have stratified analyses by age to examine rates and intent specifically for young children. Of these, one using Center for Disease Control (CDC)’s Wide-ranging Online Data for Epidemiologic Research (WONDER) database estimated the firearm death rate for 0–4 year-olds as 0.8 per 100,000 in 2021, with assault/homicide most common, and increasing by 66%, in this age group from 2018 to 2021 [13]. A study using the CDC’s National Center for Health Statistics Web-based Injury Statistics Query and Reporting System (WISQARS) database (2001–2019) examined intent by age group and found that for 0–4 year-olds, 71% of firearm injury deaths were due to assault/homicide, and 29% were unintentional [14]. The proportion of unintentional firearm injury deaths decreased stepwise with child age, from 29% at 0–4 years to 3% at 15–19 years; assault/homicide was the most commonly-classified intent for all ages [14]. An analysis of unintentional firearm injury deaths using 2003–2021 National Violent Death Reporting System (NVDRS) data, which links information from death certificates, law enforcement reports, and medical examiner records, also found that 29.1% of such deaths were among 0–5 year-olds [15]. Of studies exclusively examining 0–5 year-olds, an analysis of WISQARS data (2010–2020) found that 65.9% of 1,220 firearm deaths were classified as assault/homicide (e.g., violent interpersonal family conflicts; being a bystander) compared to 30.0% as unintentional (e.g., accidental discharge) [2]. An analysis of trends among young children using WISQARS data from 1998 to 2018 showed that unintentional firearm deaths increased exponentially among children aged 2 and 3 years old, increasing by 8.3% and 6.5% per year, respectively [16].

Considering fatal and non-fatal outcomes, National Trauma Data Bank (NTDB) data showed that approximately 5.5% of all pediatric firearm injuries in the US from 2013 to 2017 occurred among children under 6 years old [17]. Of these, 39.5% were classified as assault and 50.4% as unintentional, with toddlers—defined as 3–6 year-olds—having the highest number of unintentional injuries (*n* = 424; [17]). In this sample, 7.2% of infants (0–2 years old) and 5% of toddlers died of their injuries [17]. Another analysis of NTDB data (2007–2014) identified that among 0–4 year-olds, 20.6% of firearm injuries were unintentional, and 17.3% were intentional [18]. Taken together, studies suggest unique aspects of firearm injury patterns in younger children, with both assault/homicide and unintentional injuries increasing.

### Demographic correlates: sex and race/ethnicity

As in older children, firearm injuries among young children are not equally distributed [13, 16, 19]. Males represented 67.2% of firearm injuries among children 0–4 years old (2010–2016 NTDB data; [20]. Other studies examining sex differences in young children also found males accounted for the majority of firearm deaths among 0–5 year-olds (2010–2020 WISQARS data [2] and more male than female fatalities among 2–4 year-olds (2009–2018 NVDRS data; [3]). An analysis of WISQARS data found that unintentional firearm deaths increased exponentially among boys aged 1–4 years between 1999 and 2018 [16].

Patterns of fatal and non-fatal firearm injury have been shown to vary by race and ethnicity in the few studies that have examined this in young children. Black, non-Hispanic children (*hereafter*, Black) had the highest mortality rates in these studies [2, 19, 20]. A study of firearm deaths in 1–5 year-olds from 1976 to 2016 based on Multiple Cause-of-Death Mortality Data from the National Vital Statistics System found that the mortality rate for Black children was three times that of White, non-Hispanic (*hereafter*, White) children [19]. Analysis of NTDB data (2010–2016) found that 55.7% of 911 firearm injuries to 0–4 year-olds were among Black compared to 27.4% among White children [20]. Black children accounted for most of the increase in firearm injury deaths of 0–5 year-olds between 2010 and 2020 (WISQARS data; [2]). Another analysis of WISQARS data from 1999 to 2018 found a 4.9% yearly increase among Black children in this age group [16]. In an age-matched sample that included 0–5 year-olds as 9.8% of the sample, Hispanic (21%) and Black (77%) children had a higher likelihood of being hospitalized for firearm-related compared to motor vehicle injury as compared to White children [21]. This study did not further examine differences within the 0–5 age group, however. Taken together, findings illustrate how sex and race/ethnicity echo disparities in overall patterns of firearm injuries. Although sex and race can be considered “child factors” in the Bronfenbrenner model, they are also socially patterned and thus these findings reflect influences at the broad social-contextual levels that shape patterns of firearm injury and death even in young children. Hence, more work is needed to elucidate the root causes of these disparities (e.g., structural racism, sexism).

## Research question 2: contexts of firearm injury among children 0–5 years

### Home setting

Among 7487 children ages 0–14 years, 18% of whom were 0–4 years old, unintentional firearm injuries occurred at home 64.9% of the time, compared to 44.7% of the time in public settings (NTDB data; [18]). An analysis of NVDRS data suggested children under age 5 are more likely to be injured at home compared to children 10–14 years old (74.2% vs. 50.1%; [3]). An analysis of National Electronic Injury Surveillance System data found that as child age increased, the percent of firearm-related injuries occurring at home declined, from 63.3% for children under age 5 years, to 54.5% for children aged 6–10, to 32.4% for children aged 11–15 [22]. These statistics suggest that younger children are readily accessing firearms within their home, prompting a need for increased research surrounding firearm injury in this age group in relation to ownership, storage, and supervision.

Handgun ownership at home can be of unique concern for young children because these guns are more likely to be owned for protection and thus kept more easily accessible than long guns [23]. They are also easier for young children to operate than long guns. Research has shown that a quarter of children as young as 3 years old have the finger strength to pull the trigger of a handgun with 10 pounds of trigger-pull weight [24]; most modern handguns only require about 5–8 pounds of trigger-pull weight [25]. Handgun ownership among families with children 0–5 years old has increased over time, such that 72% of families with young children who owned firearms owned a handgun in 2016, compared to 49% of families in 1976 [19]. Although not specific to handguns or young children, findings of increases in new firearm ownership during the COVID-19 pandemic [26] could raise concerns for young children. Having children at home was a significant predictor of firearm purchases in a sample of 263 adults who purchased a firearm during the pandemic [27], and parents of older children reported increasing access to firearms in part due to fear across this period [28]. One study found that 51.6% of new firearm owners who purchased a firearm in response to COVID-19 reported having children under 5 years old at home, and that most new purchasers had done so for protection purposes [29]. A study comparing firearm-related to other injuries during the pandemic’s stay-at-home phase compared to previously found increased firearm-related injuries among 1–4 year-olds, with fewer such injuries for infants < 1 year old (NTDB data; [30]. Taken together, increasing ownership, particularly of handguns and among families who are new owners could present unique issues for firearm injury risk to young children in the home setting.

### Injury circumstances: storage and supervision

Understanding how firearms are stored and how young children can access them is essential for preventing injury. As noted above, if young children access an unlocked handgun, they are likely able to fire it. A 2018 study including children ages 0–18 years found that homes with younger children (0–5 years) were more likely to store firearms securely (locked, unloaded), but owning handguns was associated with less-secure storage [31]. A more recent nationally representative survey found that 15% of firearm owners with children of all ages at home reported storing firearms unlocked and loaded [32]. Taken together, these studies suggest many young children are likely living with firearms that are not stored securely. Yet overall, the circumstances of firearm storage and access in households with younger children are not well-characterized. The vast majority (99%) of unintentional firearm injury deaths of 0–5 year-olds from 2003 to 2021 are attributed to firearms that were stored both unlocked and loaded [15]. Although firearm type is not always known [7], when reported, handguns are responsible for most firearm-related fatalities in young children; analysis of NVDRS data found that among 1–4 year-olds, 90% of deaths were due to handguns [16]. Storing firearms—particularly handguns—locked and unloaded can likely mitigate firearm injury risk for young children.

Some research has examined detailed circumstances of firearm fatalities among young children. Of unintentional firearm injury deaths in 0–5 year-olds (NVDRS data, 2003–2021), most deaths were self-inflicted shootings (58%) by firearms that belonged to the parent of the shooter (60%; [15]). Of unintentional firearm injury deaths in 0–5 year-olds that were not self-inflicted, the shooter was most often a sibling (59%) and/or a 2–10 year-old (66%) child [15]. Playing with a firearm or showing it to others (67%) or mistaking the firearm for a toy (28%) were the most common circumstances leading to unintentional firearm injury deaths of 0–5 year-olds [15]. Analysis of locations where shooters accessed firearms found that firearms were retrieved from locations that young children can easily access such as the nightstand or bed (34%), shelf/closet (14%), or inside a vehicle (12%; [15]). Of course, not all in-home shootings are unintentional or child-inflicted. Many young children injured by firearms are victims of assault and interpersonal violence caused by adults in the home [2].

### Outside the home

Beyond the home setting, young children can be directly injured by firearms and indirectly affected by firearm-related violence. As described above, race/ethnicity disparities in firearm-related deaths are seen for young children and children can be victims of firearm violence as bystanders inside and outside the home [2]. Even when young children are not directly injured, they may witness firearm violence. Shootings can occur near child/family-friendly public locations like schools, playgrounds, and zoos [33]. A survey with caregivers of young children found that 11.5% of children 2–4 years of age had indirectly witnessed gun violence (e.g., hearing gunshots) [34].

## Research question 3: impacts of firearm injury on children 0–5 years and their families

### Mortality and physical impacts

Firearm injuries in 0-5-year-olds have devastating and far-reaching impacts. Young children are more likely to die from firearm injuries than older children [7, 20]. In almost 18,000 emergency department visits (2010–2015) researchers found that children 0–4 years old had the highest case fatality rate (15.3%); children younger than 5 were 2.7 times more likely to die compared to older children [7]. Children under 5 years face distinct challenges when recovering from gunshot wounds due to the anatomical harm caused by firearms. Given their smaller body and larger head size, young children are more likely to sustain head injuries [20], skull and face fractures [22], and also have multiple organs impacted by a gunshot wound than older children [7]. Firearm fatalities among children under age 5 years typically occur due to wounds to the head/neck (49%) and/or face (31%) [3]. A study using Virtual Pediatrics System, LLC data for approximately 1,500 firearm-related pediatric intensive care unit (PICU) cases across 135 PICUs (17% 0–5 year-olds) between January 2009 to December 2017 found no differences in survival rates within this age group, but overall, children with head and neck injuries were more likely to die from their injuries [35].

Although not focused on young children, a few studies have followed youth longitudinally after firearm injury and found ongoing long-term health impacts including complex chronic conditions [36] and pain disorders [37]. Young children who do survive their injuries may also experience ongoing physical impacts or impairments and although they may recover physically, may also lack the cognitive capacity for understanding what happened to them and why.

### Individual psychological impacts

In addition to physical impacts there can be significant psychological impacts of firearm injury for young children. Less is known about youth whose exposures occur before age 5 given limited longitudinal data [38], but in a study of 2178 youth (7.4% ages 0–5 years), over a quarter were diagnosed with a new mental health condition, primarily trauma- or stress-related, in the 12 months after non-fatal firearm injuries [39]. In a matched case-control study that included children 3–17 years old (but did not stratify by age), children had 50% increased odds of having a new mental health diagnosis in the year following a firearm injury compared to a motor vehicle crash injury [40].

Even if not directly injured, witnessing or losing a loved one due to firearm violence can create vicarious trauma that has deleterious impacts on child development [38]. One study of over 50,000 youth aged 0–19 years showed that the closer a child was to a shooting event, the more likely they were to experience increased acute mental health symptoms in the days following [41]. Although this study did not stratify by age, it is important to note that neighborhood firearm violence may cause trauma for young children by heightening feelings of stress, fear and uncertainty. Indeed, some have called for firearm violence exposure to be considered an “adverse childhood event” or ACE [8]. As the brain undergoes rapid development during early childhood, young children are especially vulnerable to the negative effects of firearm violence and can experience emotion regulation difficulties as a result [38, 42]. Little work has documented this among children under age 5, but one study found that compared to older children, children under age 10 years were more likely to be reported as ‘very’ or ‘extremely scared’ by indirect firearm violence exposures [34]. Another study found that children aged 2–9 years experienced post-traumatic stress related to hearing and witnessing gun violence (although this study also did not stratify by age) [43]. Developmentally, children under age 5 years are particularly susceptible to such exposures as this is a sensitive period not only for brain development but also for establishing safety and security in caregiving relationships [44]. When these processes are disrupted, children can experience short- and long-term psychological consequences that can contribute to a cycle of violence including anger, retaliatory attitudes, social withdrawal, and desensitization to violence [45]. Thus, while studies specifically examining long-term psychological impacts of firearm injuries in young children are scarce, research on the effects of indirect exposure suggests they could impact mental health across the lifespan.

### Family-level impacts

Few studies have examined impacts of young children’s firearm injuries on families. However, studies of older children who had experienced firearm injury have found that in the post-injury period, both survivors and family members can experience disruptive behavior, stress, anxiety, and depression and mood disorders [37, 38]. More research is needed on what happens in families when shootings involve young children. Developing trauma-informed services that address not only symptoms but also parents’ feelings of grief and guilt when a young child is shot is essential [46–48]. Symptoms of trauma can be difficult to identify in very young children and may emerge over time, so it is important to provide families with specific and ongoing supports to recognize behaviors that may signal traumatic responses in their young children. As families are the primary support systems for young children’s health and development, we also need to address challenges with obtaining services and other stressors that impact families’ capacities to support their children [49].

### Community-level impacts

Beyond individuals and families, firearm injuries and violence may heighten fear and decrease community trust. While ripple effects of firearm injuries and deaths specific to young children on communities are not well documented, such stories can be covered in the media as indicating social disorder and parent lack of responsibility [50]. Firearm violence can have negative implications for parent functioning [51] and mental health [52], which could be magnified for parents of young children and undermine their role as a protector in keeping children safe. Thus, firearm violence–perhaps particularly involving young children–can create a legacy of trauma in the most-impacted communities.

## Discussion and synthesis: developmentally-Informed strategies for prevention

A key finding of this review is that little research focuses exclusively on firearm injuries in the 0–5 year age range (see Table 1). Despite this, children under age 5 years are at increasing risk for firearm injury, with patterns by sex and race that mimic those for older children such that males and non-Hispanic Black children are most impacted. Most injuries happen at home, with unintentional injuries due to presence of unlocked, loaded firearms–often handguns–in locations that children access when even briefly unsupervised. Assault by adults in the home is common, either directly or as a bystander. Young children experience severe injuries from firearms and often die, which has ripple effects on families and communities.

Given these findings, there is an urgent need to move beyond documenting risk to consider how to prevent such injuries [53, 54]. Approaches to firearm injury prevention for children under 5 years of age must be implemented across the multilevel, interconnected child-serving ecosystem serving young children and their families [49]. Framing these findings in the bioecological model of development [10], approaches to prevention should consider individual child factors such as age and sex, and proximal processes in the microsystem of the home setting, particularly how firearms are stored, how young children are supervised, and presence of violence in the home. It is also critical to leverage mesosystem factors (e.g., connections between parents and pediatric providers) and acknowledge exosystem factors (e.g., perceptions of what other parents do) that may influence how parents approach acquiring and storing firearms. Of course, macrosystem and even chronosystem factors are also influential (e.g., geographic differences in norms around firearm ownership, historical events or cultural changes over time).

We combine this developmental approach with the Haddon Matrix for firearm injury prevention [55] to consider unintentional and intentional firearm injury prevention in early childhood, in light of the above findings. The Haddon Matrix categorizes opportunities for prevention into *hosts* (young children, the “”person” in the bioecological model), *vectors/agents* (not represented in the bioecological model), *proximal physical-social environment* (processes in the microsystem and mesosystem connections), and *broad social-contextual environment* (community norms, policies in the exo- and macrosystems) at three different stages: before the gun is fired, when the gun is fired, and after the gun is fired [55]. We focus on Haddon’s pre-injury stage, before the gun is fired, and illustrate developmentally-informed opportunities for prevention within the Haddon Matrix. Table 2 includes papers that either test interventions or review best practices for intervention at each of these levels. As in Table 1 we focus on intervention approaches focused on the 0–5 age range, considering that interventions at the broad social-contextual level apply broadly across ages.

### Host

We consider children under age 5 as the “host”. As siblings are often involved in shootings [3, 15, 56], host-level considerations can also apply to siblings. At the *host level*, some have tried teaching children directly about the risks of touching a firearm. Small studies tested behavioral skills training interventions with ages 4–5 years [57, 58]. Such interventions are resource-intensive and have had only mixed success. Children also respond differently when asked about a hypothetical scenario (“what would you do if you saw a gun”) compared to an in-situ scenario involving a realistic hidden firearm [59]. Given the ease of use (especially for handguns) and lethality of firearm injury to young children, this difference is concerning. One study found that 3–7 year-olds were less likely to touch a firearm after a parent-mediated online training, but the sample was very small (*n* = 9) [60].

Although children under 5 are likely too young to engage in formal firearm safety training, it is important to understand how child development factors may shape behavior around firearms. Being male is identified as a risk factor perhaps in part driven by child impulsivity [61] as well as environmental factors (e.g., modeling through videogames [62]). In general, young children are more likely to be injured when parents either over- or under-estimate their abilities [63]; accurately characterizing children’s early developmental capacities may help protect from firearm injury. From infancy, children are naturally curious, drawn to novelty, and actively explore [64, 65]. Children under 2 years old can find hidden objects [66] and manipulate items similar in size to small firearms [67]. As noted above, many 3-year-olds have the finger strength to fire a handgun [24] and firearm injury rates increase among 2- and 3-year-olds [16]. Children’s ability to remember where things are hidden also increases dramatically from 2 to 3 years old [68]. Thus, young children may be interested in firearms, seek them out, and manipulate them, but may not have the cognitive or impulse control to refrain from touching them even if told about safety rules.

### Agent

At the *agent level*, prevention can involve modifying firearm design and/or storage in ways that decrease risk to young children, for example through personalization of locks or fingerprint technology. Proximity devices such as radio frequency identification (RFID)-enabled bracelets, rings, or watches worn by a parent can also make it less likely for young child to fire a weapon [54], although these may not appeal to all due to trust in the reliability of RFID technology and/or cost concerns [69]. Many different locked storage options exist, from cable locks, to gun cases or lock boxes, to safes. Young children are unlikely to be able to bypass these directly. Yet, if a storage device is locked by key or access code and children (or their siblings) know the code or where the key is hidden, they may be able to open the device and access the firearm, particularly as manual dexterity and working memory improve. Finally, although cable locks are often available for free, smart-tech options are more costly [69]. For families who primarily own firearms for protection (e.g., who perceive their neighborhood as unsafe), qualitative work has shown the importance of secure but quick access, a characteristic of smart guns or smart safes [70, 71]. Agent-based strategies that are effective and allow quick access may encourage individuals who perceive their environment to be unsafe to engage in secure storage, as such beliefs and protection motivation predict firearm ownership and storage behavior [72–74]. To prevent firearm injury to young children, it is important to identify whether tech- or other agent-based solutions may meet these needs, and if so make them available, affordable, and appealing for families with young children.

### Proximal physical/social environment

Parents fundamentally shape a young child’s *proximal physical and social environment.* They can play an essential role in keeping the physical home microsystem environment safe by securely storing firearms locked and unloaded. Some surveys report that parents of children of all ages are more likely than non-parents to keep firearms stored locked and unloaded [74, 75]. Yet, parents also cite protection as a primary motivation for owning firearms [74], and thus may store their firearms loaded and unlocked in case they need to be quickly accessed for self-defense [70, 74]. Less is known regarding firearm storage and access beliefs and practices specifically in households with younger children. Educating about secure storage methods in addition to making gun safes and locking devices faster and cheaper or free may increase utilization [76]. In particular, parents who are new firearm owners may know less about storage methods.

Safety in the home microsystem includes not only secure firearm storage but modeling safe behaviors when using firearms and supervising young children if firearms are in the home. Understanding how and when parents use firearms, how children are supervised, and rules and routines are important for creating safe physical and social environments. For example, parents who need to transport young children and firearms need options to store safely in vehicles. In contrast, parents of young children who own firearms for collecting or sporting purposes may consider storing outside the home [54]. Establishing clear routines and expectations around firearm-related activities is critical. Yet, parenting young children is challenging and parents face competing needs [49]. It is important to support parents, especially those facing challenges like depression, substance abuse, interpersonal or community violence to create safe and predictable home environments. Programs designed to reduce firearm violence may help. For example, although these take place after an initial firearm injury has occurred, Hospital Violence Intervention Programs (HVIP) may help motivate parents to prevent future injury, at teachable moments [77]. As most HVIPs focus on young adults, they provide a model for engaging youth who may already be or may soon become parents of young children in the importance of promoting firearm safety behaviors and violence prevention.

Outpatient counseling of parents around firearm storage during pediatric visits is an example of a mesosystem interaction between parent and provider; when parents and providers connect, it can promote positive child outcomes. Although firearm storage counseling can increase parents’ secure storage practices [78], it does not occur frequently enough [79]. Screenings followed by interventions like motivational interviewing can further increase secure firearm storage [80]. Although many parents think pediatricians should advise about safe storage of firearms (up to 71% of firearm owners; [81]), the framing is critical, with parents also reporting they would ignore advice not to have household firearms [81], and qualitative work revealing strong parent preferences for privacy and fear of government overreach [82]. Given the large number of pediatric visits during the early childhood years covering anticipatory guidance like babyproofing to avoid injury, it is critical that pediatricians are trained in culturally competent communication and encouraged to engage around this important issue [79, 83]. As an additional support in this setting, lighter-touch counseling methods using technology-based interventions around anticipatory guidance on firearm safety (e.g., on a tablet in the waiting room) may also be less threatening or could open the door to a conversation with the provider [84].

### Broad social-contextual

There are multiple approaches for preventing firearm injury to young children at the *social-contextual level* through changing behaviors, community norms, programs, or policies in the exo- and macrosystems. Strategies that seek to change parent behaviors specific to firearms include campaigns like Asking Saves Kids (ASK) and Be SMART that promote caregivers asking if there is a firearm where their child spends time outside of home and how it is stored [85]. This could start as soon as a child may spend time in places within the parents’ exosystem (e.g., family friends, grandparents). If started early, this “asking habit” early could change individual parent behavior and help shift community norms by normalizing asking if a firearm is present and stored properly. Credible community messengers who share identities as parents of young children who also own firearms are a promising way to deliver such messages [86]. Media messages that highlight the importance of secure storage starting in early childhood are another way to change social norms around firearm injury prevention [87].

More broadly, community-based efforts to decrease firearm violence and injury may prevent injury to young children. Strategies that improve the built and social environments can impact community-level connectedness and firearm violence [88]. Evidence suggests that gun violence may be mitigated by community-based gun buyback and violence prevention programs [89]. Community violence intervention programs seek to break the cycle of violence by using frontline workers with community credibility. Such efforts may have long-lasting impact if they reduce exposures during early childhood, when the impacts of stress and violence exposures can be most detrimental and shape behavioral and health outcomes over the lifespan, including long-term epigenetic modifications, which can transmit across generations [90].

Finally, legislative policies may help prevent firearm injury to young children. Unintentional firearm death rates in children 1–4 years old were positively associated with handgun permits issued and firearm background check rate [16]. Child access prevention laws (CAP) vary by state and are viewed as a legislative method of making firearms less accessible to children [56]. In an ecological, cross-sectional study of CAP laws between 1991 and 2016, more stringent CAP laws were associated with statistically significant reductions in firearm fatalities in children aged 0–14 years, although this analysis was not further stratified by age [91]. Unsafe storage was also less likely in states with CAP laws and strong firearm legislation [92]. A more recent study identified limited effects of CAP laws on storage, and most gun owners reported being unaware that CAP laws existed in their state [93]. Continuing to evaluate CAP laws and the implementation and education around these and other laws [94] specific to firearm injury among young children will be important.

## Conclusions, limitations, and recommendations

This critical review provided a developmentally-informed overview of key issues regarding firearm injuries among children under age 5 years. The prevalence of firearm injuries in this age group has risen in part due to increasing numbers of new firearm owners, a trend that does not appear to be stopping. The direct and indirect impacts of firearm injury to a young child are significant and long-lasting, yet preventable. Our review revealed a gap in research specifically examining firearm injuries in the early childhood period. Collecting more extensive data on incidents involving children under 5 years of age is necessary to better understand the contexts and precursors of firearm injuries at home and outside-of-home settings, and support the promotion of multi-level, developmentally-informed prevention strategies.

Limitations of this work were that we elected to conduct a critical review rather than a systematic or scoping review of the literature, which necessarily means a more subjective interpretation [9]. The critical review process is also less systematic than other more structured review formats. The firearm injury literature is highly interdisciplinary, so we may have missed some literatures with relevant information. Some of the analyses discussed were based on the same datasets, which is generally the state of the literature, but there could be bias due to the availability of data and systematic missing data on injury details for children in this age range.

We recommend that more studies compare firearm injury data across ages with regard to rates, intent, and outcome (e.g., fatal vs. non-fatal injury), and call for more research specifically on the 0–5 age group in order to characterize and understand firearm injury burden in early childhood. As these firearm injuries remain statistically rare, conducting qualitative and mixed-methods studies can also inform our understanding, particularly around prevention. Such efforts must include community members who are parents of young children and who own firearms, and those impacted by gun violence [95]. It is also critical to attend to disparities in the patterns and contexts of injury; similar to other age groups, males and Black children in the 0–5 age range experience higher rates of firearm injury and death. Finally, although more national and longitudinal data are being collected, given reduced restrictions on funding for firearms-focused research most data remain correlational. Leveraging natural experiment opportunities to assess impact of policy changes and other interventions on firearm injuries among young children will be crucial in moving the science of pediatric firearm injury prevention forward.

## Data Availability

No datasets were generated or analysed during the current study.

## Abbreviations

ASK: Asking saves kids
ACE: Adverse childhood experience
CDC: Centers for disease control and prevention
CAP: Child access protection
HVIP: Hospital violence intervention programs
NTDB: National Trauma Data Bank
NVDRS: National violent death reporting system
PICU: Pediatric intensive care unit
RFID: Radio Frequency IDentification
WISQARS: Web-based injury statistics query and reporting system
WONDER: Wide-ranging online data for epidemiologic research
